# The IRE1α pathway in glomerular diseases: The unfolded protein response and beyond

**DOI:** 10.3389/fmmed.2022.971247

**Published:** 2022-09-26

**Authors:** José R. Navarro-Betancourt, Andrey V. Cybulsky

**Affiliations:** Department of Medicine, McGill University Health Centre Research Institute, McGill University, Montreal, QC, Canada

**Keywords:** autophagy, cell injury, endoplasmic reticulum, glomerulonephitis, podocyte

## Abstract

Endoplasmic reticulum (ER) function is vital for protein homeostasis (“proteostasis”). Protein misfolding in the ER of podocytes (glomerular visceral epithelial cells) is an important contributor to the pathogenesis of human glomerular diseases. ER protein misfolding causes ER stress and activates a compensatory signaling network called the unfolded protein response (UPR). Disruption of the UPR, in particular deletion of the UPR transducer, inositol-requiring enzyme 1α (IRE1α) in mouse podocytes leads to podocyte injury and albuminuria in aging, and exacerbates injury in glomerulonephritis. The UPR may interact in a coordinated manner with autophagy to relieve protein misfolding and its consequences. Recent studies have identified novel downstream targets of IRE1α, which provide new mechanistic insights into proteostatic pathways. Novel pathways of IRE1α signaling involve reticulophagy, mitochondria, metabolism, vesicular trafficking, microRNAs, and others. Mechanism-based therapies for glomerulopathies are limited, and development of non-invasive ER stress biomarkers, as well as targeting ER stress with pharmacological compounds may represent a therapeutic opportunity for preventing or attenuating progression of chronic kidney disease.

## Introduction

A large body of evidence from both preclinical studies and human samples indicates that endoplasmic reticulum (ER) function is important for protein homeostasis (“proteostasis”) in the kidney, and that ER stress due to ER protein misfolding and/or calcium depletion is a component of various kidney diseases (Inagi et al., 2014; Cybulsky, 2017; Ricciardi and Gnudi, 2020; Gomez-Sierra et al., 2021; Li and Chen, 2021). Among these are glomerular diseases that are associated with injury to podocytes. ER stress in glomerulopathies is linked with the activation of the unfolded protein response (UPR) and autophagy (Cybulsky, 2017). Inositol-requiring enzyme 1α (IRE1α) is the most multifaceted transducer of the UPR and it has received considerable attention in recent years. This review provides an overview of proteostasis and ER stress, with a focus on the glomerulus, and highlights recent developments in the field based on experimental models and human studies. In particular, we emphasize the role of IRE1α in the UPR and autophagy (reticulophagy), and describe novel pathways of IRE1α signaling involving mitochondria, metabolism, vesicular trafficking, microRNAs, and others. There is also a brief discussion of an emerging area of research involving ER stress biomarkers and approaches to therapeutics.

## The podocyte in health and disease

Podocytes, or glomerular visceral epithelial cells (GECs), are terminally-differentiated cells that have a highly organized cytoskeleton and possess contractile foot processes that stretch along the exterior surface of the glomerular basement membrane (GBM) (Reiser and Altintas, 2016). Podocytes, together with fenestrated glomerular endothelial cells and the GBM, form the glomerular filtration barrier, a multilayered functional structure that allows the filtration of water, solutes and toxins, but restricts cells and plasma proteins (Fissell and Miner, 2018). The foot processes of neighboring podocytes interdigitate and connect through filtration slit diaphragms that constitute the final barrier to blood filtration and confer glomerular permselectivity (Kawachi et al., 2006). The slit diaphragm is a multiprotein structure that resembles an adherens junction (Greka and Mundel, 2012); however, the slit diaphragm is more than an intercellular adhesion complex and a molecular sieve, since it is also a signaling hub that integrates mechanical and chemical stimuli from the subepithelial space, and communicates signals that regulate the intricate cytoskeletal structures of multiple podocytes (Grahammer et al., 2013). Podocyte function depends on highly coordinated processes, including the preservation of an intricately organized cytoskeleton and it involves production of complex structural proteins of the slit diaphragm, adhesion complexes, and the GBM (Reiser and Altintas, 2016). Since, podocytes have limited regenerative capacity (Greka and Mundel, 2012), they are uniquely sensitive to injury, and maintenance of proteostasis is critical. Thus, podocytes must adapt to multiple intrinsic or extrinsic perturbations in the context of aging and disease (Puelles et al., 2016).

Injury and/or loss of podocytes is the hallmark of human glomerular diseases, such as focal segmental glomerulosclerosis (FSGS), membranous nephropathy and diabetic nephropathy (Assady et al., 2017; Woo et al., 2019). These glomerulopathies often progress to chronic kidney disease (CKD) (Kriz and LeHir, 2005; Kriz et al., 2013), which is associated with increased mortality and imposes a serious public health burden (Hill et al., 2016; Glassock et al., 2017). Current treatments of glomerular diseases are only partially effective, significantly toxic, lack specificity, and are often insufficient to prevent end-stage renal failure (Romagnani et al., 2017). Thus, it is important to develop mechanism-based therapies to halt or attenuate glomerulopathies.

Idiopathic FSGS is believed to be caused by an unknown circulating factor that is toxic to podocytes (Rosenberg and Kopp, 2017), while in membranous nephropathy, podocytes are attacked by autoantibodies (Couser, 2017), in most cases directed against the phospholipase A2 receptor in the plasma membrane (Cattran and Brenchley, 2017). These antibodies activate complement, and the assembly of C5b-9 in podocyte plasma membranes leads to cell damage (Couser, 2017). In diabetic nephropathy (which affects 30–40% of diabetic patients and is the most common cause of CKD (Alicic et al., 2017)), multiple factors from the diabetic microenvironment contribute to podocyte injury, including hyperglycemia, growth factors, inflammatory cytokines, and glomerular hyperfiltration (DeFronzo et al., 2021). In many cases, these glomerular diseases lead to nephrotic range proteinuria and nephrotic syndrome (Kopp et al., 2020). FSGS may also result from mutations in genes encoding components of the glomerular filtration barrier (Lipska-Zietkiewicz et al., 2020), including slit diaphragm proteins such as nephrin (Lenkkeri et al., 1999) and podocin (Bouchireb et al., 2014), cytoskeletal components (Kaplan et al., 2000), mitochondrial proteins (Doleris et al., 2000; Gucer et al., 2005), and structural elements of the GBM, such as laminin-β2 (Zenker et al., 2004) and type IV collagen (Kruegel et al., 2013).

Acquired podocyte injury may be caused by degenerative, toxic, or inflammatory processes (Kriz and LeHir, 2005); the common outcomes of these mechanisms are foot process effacement, attenuation of the cell body, pseudocyst formation, and cell detachment, leaving areas of naked capillary loops that fail to retain plasma proteins (macromolecules) (Pavenstadt et al., 2003; Reiser and Altintas, 2016). Apoptosis of podocytes has been demonstrated in cell culture studies using various conventional techniques, while apoptosis *in vivo* has been reported in rodent models, mainly based on tissue staining with terminal deoxynucleotidyl transferase-mediated dUTP nick end-labeling (TUNEL) or caspase-3 staining. Interestingly, apoptosis has not been observed in human podocytes by electron microscopy (Kriz et al., 2013; Nagata, 2016; Yin et al., 2021). These discrepant results between rodents and humans may be in part related to experimental techniques, or may represent species differences. Thus, podocyte loss is probably a consequence of detachment of viable cells (Kriz et al., 2013; Nagata, 2016; Yin et al., 2021). After initial injury, it is believed that remaining podocytes widen their foot processes and undergo hypertrophy in an attempt to maintain the glomerular filtration barrier, and this additional workload accelerates podocyte loss (Kriz et al., 2013). Ultimately, ongoing damage to the podocyte network cannot be repaired and leads to nephron degeneration. In the absence of podocytes, the naked capillary tuft adheres to the glomerular capsule and segmental sclerosis ensues, which eventually leads to obliteration of the glomerulus (Kriz and LeHir, 2005).

Importantly, cells may respond to injury by activating adaptive pathways that limit injury or promote recovery. A detailed understanding of such pathways activated in podocytes in the course of aging and glomerular diseases is necessary to develop mechanism-based interventions that protect podocytes and preserve glomerular permselectivity (Cybulsky, 2017). An increasing amount of evidence has demonstrated that in podocytes—long-lived cells that face a high secretory workload—the function of the ER is compromised in multiple glomerular diseases, and targeting the ER may provide therapeutic opportunities (Cybulsky, 2013, 2017; Gomez-Sierra et al., 2021; Li and Chen, 2021).

## UPR

Secreted and membrane-bound proteins constitute one third of the cellular proteome. These proteins are processed in the ER, where they are covalently modified with the assistance of folding enzymes and chaperones, allowing them to reach their native conformation (Wang and Kaufman, 2016). Protein synthesis is inherently error-prone; in fact, 15% of average-length proteins have at least one mis-incorporated amino acid (Drummond and Wilke, 2009). Moreover, post-translational modifications are not mRNA template-driven and are thus more susceptible to variation (Nilsson et al., 2013). To maintain proteostasis, cells have developed quality control systems that adjust protein folding, trafficking, and degradation; together, these resources constitute the cellular proteostasis network (Hipp et al., 2019).

**FIGURE 1:**
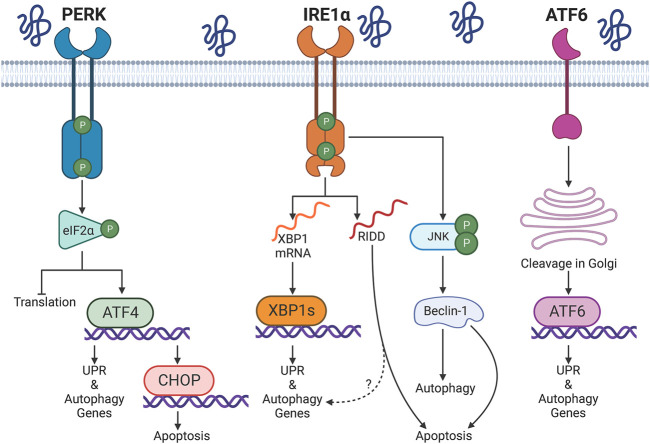
Overview of the unfolded protein response (UPR). Protein misfolding in the endoplasmic reticulum (ER) activates three UPR transducers: inositol-requiring enzyme 1α (IRE1α), protein kinase R-like ER kinase (PERK), and activating transcription factor 6 (ATF6). PERK phosphorylates eukaryotic initiation factor 2α (eIF2α); this attenuates cap-dependent translation and allows upregulation of activating transcription factor 4 (ATF4). IRE1α splices X-box binding protein 1 (XBP1) mRNA, which produces the transcription factor XBP1s. Multiple mRNAs or miRNAs are degraded by regulated IRE1-dependent decay (RIDD). IRE1α-dependent phosphorylation of c-Jun N-terminal kinase (JNK) has been associated with activation of autophagy or apoptosis. ATF6 transits to the Golgi apparatus, where it is sequentially processed by the site-1 and site-2 proteases. Cleaved ATF6 enters the nucleus to mediate transcription. XBP1s, ATF4, and ATF6 drive a transcriptional program that upregulates the UPR, autophagy and other proteostasis resources. The transcription factor CCAAT/enhancer binding protein homologous protein (CHOP) induces a pro-apoptotic program during prolonged activation of the UPR. Graphics created with BioRender software.

The UPR is a signal transduction pathway activated by the accumulation of misfolded proteins in the ER lumen or ER calcium depletion (Hetz et al., 2020) (Figure 1). The UPR is regulated by three transducers in the ER membrane: IRE1α, protein kinase R-like ER kinase (PERK) and activating transcription factor 6 (ATF6). Well-studied actions of the UPR include upregulation of endogenous ER chaperones (to enhance protein folding), attenuation of mRNA translation (to reduce the protein load to a damaged ER), and degradation of misfolded proteins through macroautophagy (hereafter autophagy) or ER-associated degradation (ERAD), which involves the ubiquitin-proteasome system (Hetz et al., 2015). These functions are generally adaptive or cytoprotective. However, a sustained UPR can lead to an apoptotic response, e.g., via PERK-mediated activation of the transcription factor C/EBP homologous protein (CHOP) (Hetz et al., 2020) or IRE1α-dependent pathways (see below). Factors that regulate the switch from an adaptive to a pro-apoptotic UPR are under study (You et al., 2021), but remain poorly understood.

Interestingly, the UPR was discovered as a response to forced protein secretion. The first evidence of upregulation of the ER chaperones GRP78 (BiP/HSPA5) and GRP94 (endoplasmin) comes from a study in fibroblasts transformed by the Rous sarcoma virus (Shiu et al., 1977). Over the course of the following decade new research revealed that these proteins were, in fact, upregulated by stimuli that compromise protein folding in the ER (Kozutsumi et al., 1988). Most of the research revolving around the UPR has focused on terminally-differentiated cells, which need to function over long periods of time, such as neurons, or professional secretory cells, including pancreatic acinar and β-cells, hepatocytes, and plasma cells (Hetz, 2012).

Many mechanistic studies of the UPR in cell culture have used small molecules to disrupt protein folding in the ER. Classic ER perturbagens include tunicamycin, thapsigargin, and brefeldin-A, which inhibit N-linked glycosylation, deplete ER calcium, and block ER-Golgi anterograde transport, respectively (Hetz, 2012; Karagoz et al., 2019). Pharmacological induction of ER stress may overwhelm the proteostasis network and simultaneously activate the three branches of the UPR (Shoulders et al., 2013). Recent evidence suggests that the three UPR branches are differentially activated in diseases (Ordonez et al., 2013; Scaiewicz et al., 2013; Madhusudhan et al., 2015); thus, an accurate characterization of individual UPR transducers should be done in a cell type and context-specific fashion. Comprehensive reviews describing UPR activation and regulation are available (Wang and Kaufman, 2016; Hetz and Papa, 2018; Hetz et al., 2020); this work will focus on IRE1α and glomerular diseases.

## IRE1α pathway

IRE1α drives the most evolutionarily conserved arm of the UPR. The protein has a kinase and a ribonuclease domain that upon activation may mediate distinct outcomes (Ron and Walter, 2007) (Figure 1). IRE1α functions in homodimers that interact through discontinuous dimerization in both domains (Lee et al., 2008). Upon accumulation of misfolded proteins in the ER lumen, IRE1α dimers transautophosphorylate, homomultimerize, and reorganize from a face-to-face configuration into a back-to-back disposition that allows endoribonuclease activity (Ali et al., 2011). The role of autophosphorylation and the precise phosphorylation sites have not been established conclusively; however, certain studies have indicated that phosphorylation increases endoribonuclease activity (Ali et al., 2011; Prischi et al., 2014). The IRE1α ribonuclease selectively removes a 26-nucleotide intron in X-box binding protein-1 (XBP1) mRNA; spliced XBP1 mRNA encodes a transcription factor (XBP1s) that translocates into the nucleus to activate transcription of ER chaperone and autophagy genes. This enhances ER folding capacity and may promote autophagy (Yoshida et al., 2001). In addition, the IRE1α ribonuclease can degrade multiple RNAs with structures and sequences similar to the XBP1 splicing site in a process called regulated IRE1-dependent decay (RIDD); however, degradation of mRNAs through RIDD is not necessarily associated with ER stress (Maurel et al., 2014).

While XBP1 splicing is essentially adaptive, IRE1α signaling may also be pro-apoptotic, involving transcriptional and post-translational mechanisms (Chen and Brandizzi, 2013). The ribonuclease activity of IRE1α can promote cell death through RIDD; indeed, persistent activation of RNA splicing may lead to the degradation of anti-apoptotic miRNAs that normally suppress the transcription of caspase-2 (Upton et al., 2012). Moreover, under sustained ER stress, IRE1α can form a complex with tumor necrosis factor receptor associated factor 2 (TRAF2) and apoptosis signal-regulating kinase 1 (ASK1). This leads to phosphorylation of c-Jun N-terminal kinase (JNK) (Urano et al., 2000; Nishitoh et al., 2002), which produces an increase in the pool of BH3-only proteins and subsequent mitochondrial outer membrane permeabilization by Bax or Bak (Iurlaro and Munoz-Pinedo, 2016). Interestingly, accumulation of phospho-JNK and apoptosis (TUNEL staining and cleaved caspase-3) in glomeruli are enhanced in the context of XBP1 knockout (KO) in mice (Hassan et al., 2016), suggesting that when the transcriptional upregulation of proteostasis components fails, IRE1α may promote apoptosis by post-translational mechanisms.

## Autophagy and reticulophagy

**FIGURE 2:**
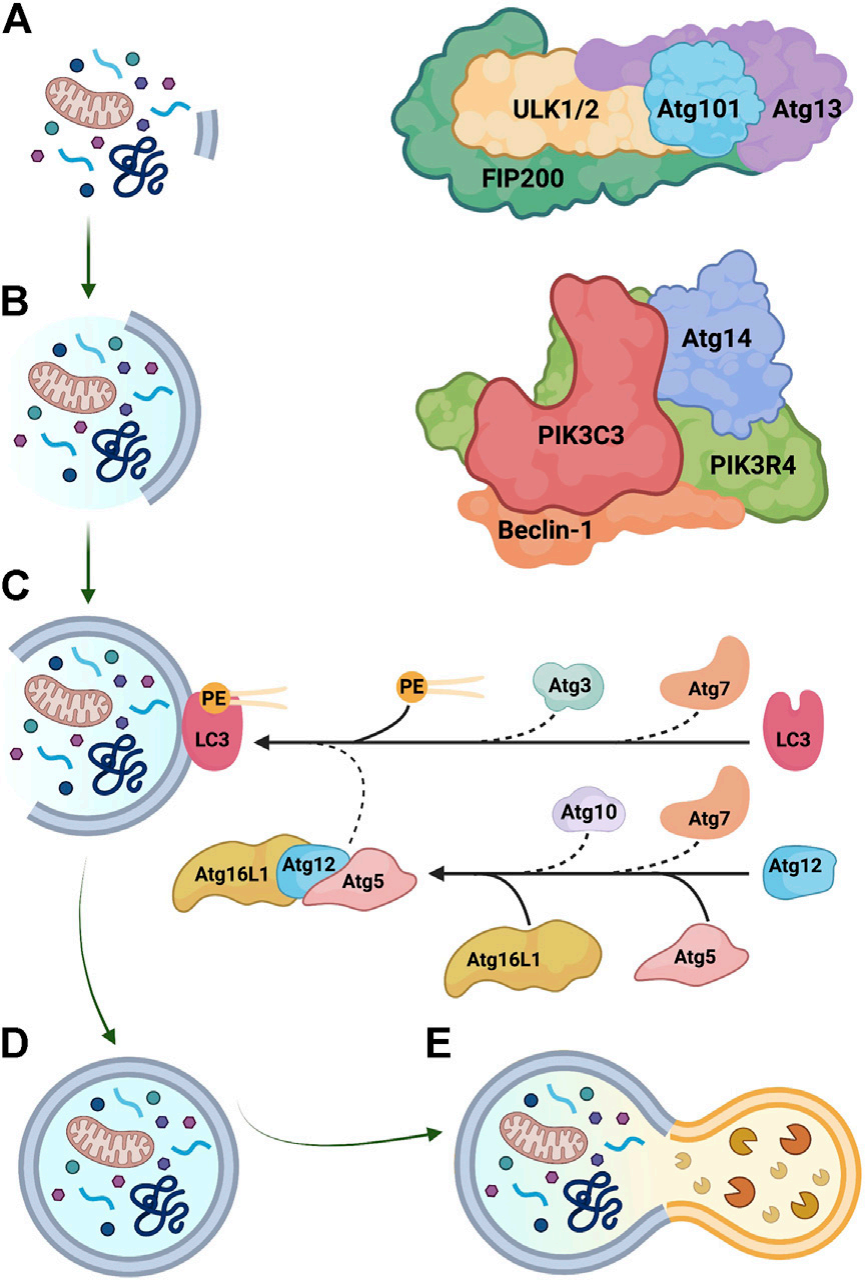
Key steps in autophagosome biogenesis. **(A)** Initiation is regulated by the Unc-51 like autophagy activating kinase (ULK) complex, composed of ULK1/2, focal adhesion kinase family kinase-interacting protein of 200 kDa (FIP200), Atg13, and Atg101. **(B)** Nucleation is mediated by the class III phosphatidylinositol 3-kinase (PtdIns3K) complex, which includes PtdIns3K catalytic subunit type 3 (PIK3C3), PtdIns3K regulatory subunit 4 (PIK3R4), beclin-1, and Atg14. **(C)** Elongation is controlled by ubiquitin-like conjugation reactions. Atg7 functions as an E1-like activating enzyme, while Atg10 and Atg3 serve as E2-like conjugating enzymes. The Atg5-Atg12-Atg16L1 has E3-like ligase activity and assists in the conjugation of microtubule-associated protein light chain-3 (LC3) to phosphatidylethanolamine (PE). **(D)** Mature autophagosomes are double-membrane organelles that contain diverse cellular components, including misfolded proteins and damaged organelles. **(E)** Autophagosomes fuse with lysosomes and contents are digested by hydrolytic enzymes. Graphics created with BioRender software.

Autophagy is a proteostasis mechanism that degrades long-lived proteins and organelles (Figure 2). The process, which is intimately related to the ER membrane (Hayashi-Nishino et al., 2009), involves the sequestration of cytoplasmic components into a double-membrane vesicle (i.e. autophagosome) that subsequently fuses with a lysosome (i.e. phagolysosome), exposing the content to hydrolytic enzymes (Boya et al., 2013). Autophagosome biogenesis comprises three phases: initiation, nucleation, and elongation (Parzych and Klionsky, 2014). In mammalian cells, initiation of autophagy is controlled by the Unc-51 like autophagy activating kinase (ULK) complex, which includes ULK1/2, focal adhesion kinase family kinase-interacting protein of 200 kDa (FIP200), Atg13, and Atg101 (Alharbi et al., 2021; Cao et al., 2021) (Figure 2A). Nucleation of the nascent autophagosome (i.e., phagophore) is regulated by the class III phosphatidylinositol 3-kinase (PtdIns3K) complex, consisting of PtdIns3K catalytic subunit type 3 (PIK3C3), PtdIns3K regulatory subunit 4 (PIK3R4), beclin-1, and Atg14 (Figure 2B) (Parzych and Klionsky, 2014). Importantly, beclin-1 is the scaffold of the PtdIns3K complex and multiple binding partners of beclin-1 modulate autophagy at this stage (Kang et al., 2011); for instance, B-cell lymphoma 2 (Bcl-2) sequesters free beclin-1 and inhibits autophagy (Decuypere et al., 2012).

Elongation of autophagosomes is mediated by two ubiquitin-like conjugation systems (Figure 2C) (Parzych and Klionsky, 2014; Dikic and Elazar, 2018). The E1-like activating enzyme Atg7 and the E2-like conjugating enzyme Atg10 produce the Atg5-Atg12-Atg16L1 complex that has E3-like ligase activity and allows the expansion of the autophagosome membrane (Mizushima et al., 2003; Boya et al., 2013; Noda et al., 2013). Through the second conjugation system, cytosolic microtubule-associated protein light chain-3 (LC3; Atg8 in yeast) is recruited to the autophagosomal membrane and conjugated to phosphatidylethanolamine by Atg7, the E2-like enzyme Atg3, and the Atg5-Atg12-Atg16L1 complex (Ichimura et al., 2000; Cao et al., 2021). Lipidated LC3 (LC3-II) is the single best marker of autophagosome biogenesis (Klionsky et al., 2021). Sequestosome-1 (SQSTM1/p62) is an adaptor protein that transports substrates into autophagosomes and is subsequently degraded with the rest of the cargo; consumption of p62 is therefore an indicator of autophagic flux and may be useful to monitor autophagy *in vivo* (Seibenhener et al., 2007). Autophagy mediators are usually found in excess in the cytoplasm, but when autophagy is sustained over a long time-frame, certain mediators may become rate-limiting; thus, transcriptional upregulation of autophagy proteins is particularly important in this context and contributes to the regulation of autophagy (Di Malta et al., 2019).

ER-resident proteins participate in autophagosome formation (Morel, 2020), and autophagy is increasingly recognized as a protein degradation resource during ER stress (Qi and Chen, 2019). Selective autophagy of the ER (reticulophagy/ERphagy) is a novel regulator of ER structure and function in mammalian cells (Beese et al., 2019; Hubner and Dikic, 2020). Reticulophagy is mediated by ER-resident reticulophagy adaptors (Grumati et al., 2018). These proteins have at least one Atg8-interacting motif (Wilkinson, 2020), which recognizes LC3 or Atg8-family proteins in autophagosome membranes and allows the delivery of ER fragments to autophagosomes (Noda et al., 2010; Hubner and Dikic, 2020). Reticulophagy adaptors are differentially distributed across ER sheets and tubules (Grumati et al., 2017; Yang et al., 2021), which suggests that these receptors have distinct cargo specificities (Chino and Mizushima, 2020). Interestingly, some reticulophagy adaptors are reported to be upregulated during the UPR (Zielke et al., 2020; Molinari, 2021). Reticulophagy assists in protein quality control (Cunningham et al., 2019; Forrester et al., 2019), and a recent report associated the IRE1α axis to activation of reticulophagy in yeast (Zhao et al., 2020). However, the role of IRE1α and physiologic implications of reticulophagy in mammalian cells are not well-understood.

The three UPR branches may participate in the regulation of autophagy (Rashid et al., 2015; Cybulsky, 2017) (Figure 1). Specifically, IRE1α can modulate autophagy via transcriptional and post-translational mechanisms. During ER stress, IRE1α mediates the phosphorylation/activation of JNK by ASK1. Active JNK phosphorylates Bcl-2, dissociating it from beclin-1; this increases the pool of free beclin-1 and allows assembly of the phagophore nucleation complex (Urano et al., 2000; Ogata et al., 2006). Of note, a transient increase in Bcl-2 phosphorylation disrupts the interaction with beclin-1 and promotes autophagy, but does not change the amount of Bcl-2 that restrains Bax, a pro-apoptotic mediator of mitochondrial permeabilization (Wei et al., 2008; Bonora et al., 2020). Thus, the degree of Bcl-2 phosphorylation by phospho-JNK may function as a molecular switch to promote adaptive autophagy or induce apoptosis (Wei et al., 2008). Additionally, phospho-JNK favors the nuclear translocation of c-Jun, and this transcription factor upregulates the expression of the beclin-1 gene (BECN1) (Li et al., 2009; Jia et al., 2014).

The IRE1α-dependent transcription factor XBP1s stimulates the expression of BECN1 (Margariti et al., 2013) and Atg3 (Sharma et al., 2017). It is important to note that XBP1s can heterodimerize with other transcription factors, including ATF6 (Yamamoto et al., 2007; Shoulders et al., 2013) and Forkhead Box O1 (FoxO1) (Zhou et al., 2011b; Kishino et al., 2017); therefore, the transcriptional scope of XBP1s should be assessed in a cell type-specific fashion. Transcriptional targets of IRE1α-XBP1s have been recently defined in podocytes (see below).

## UPR in human glomerular diseases

Activation of the UPR is evident in multiple human proteinuric diseases (Cybulsky, 2017; Chen et al., 2021). Most studies are limited by relatively small sample sizes and typically focus on the upregulation of downstream elements of the UPR, which does not allow for dissection of UPR pathways. Studies of hereditary nephrotic syndromes (and analogous experimental models) have established an association between protein misfolding in podocytes and glomerular injury (Table 1). Alport syndrome and thin basement membrane nephropathy are caused by mutations in the genes encoding the α3-5 chains of type IV collagen (COL4A3, COL4A4, COL4A5) (Kruegel et al., 2013). Activation of the UPR was evident in two heterozygous carriers of a mutation in the COL4A3 gene, as shown by increased glomerular staining for the ER chaperone BiP (Pieri et al., 2014). In cultured GECs, mutant α3 collagen IV chains are not secreted, but are retained in the ER and induce upregulation of UPR transcripts, including BiP, CHOP, and XBP1s. Mice with mutant COL4A3 developed an Alport-like phenotype, together with upregulation of XBP1s and BiP (HSPA5) mRNA levels in glomeruli (Pieri et al., 2014).

**TABLE 1:** Gene mutations predisposing to experimental glomerular diseases associated with ER stress or the UPR.

Mutated gene	Protein	Disease	Reference
LAMB2	laminin-β2	CNS^a^, Pierson syndrome	Chen et al. (2013)
			Kim et al. (2016)
NPHS1	nephrin	CNS	Drozdova et al. (2013)
NPHS2	podocin	FSGS	Ohashi et al. (2003)
COL4A3	collagen-α3	Alport	Pieri et al. (2014)
COL4A1	collagen-α1	Proteinuria, Bowman’s capsule defects	Jones et al. (2016)
ACTN4	α-actinin-4	FSGS	Yee et al. (2018)
LEPR^b^	leptin receptor	diabetic nephropathy	Shahzad et al. (2021)

a Congenital nephrotic syndrome.

b db/db mouse.

Mutations in the nephrin gene (NPHS1) cause congenital nephrotic syndrome of the Finnish type (Lenkkeri et al., 1999; Wartiovaara et al., 2004). In cultured GECs, mutant nephrin is retained in the ER and triggers the UPR (Drozdova et al., 2013). Patients with Pierson syndrome (microcoria-congenital nephrosis) have mutations in the gene encoding laminin-β2 (LAMB2), a protein in the GBM (Zenker et al., 2004). Mutant laminin-β2 is retained in the ER and activates the UPR in cultured cells (Chen et al., 2013); furthermore, mice expressing a mutant laminin-β2 transgene in podocytes showed glomerular injury (glomerulosclerosis, GBM widening, foot process effacement), ER distension and upregulation of glomerular ER chaperones and CHOP transcripts (Chen et al., 2013; Kim et al., 2016).

Analysis of gene expression in glomeruli of human kidney biopsies supports the view that the UPR is activated in FSGS. Compared to healthy living kidney donors, patients with FSGS showed upregulation of multiple genes associated with the UPR. A principal component analysis of glomerular gene expression distinguished FSGS patients from controls. Furthermore, gene ontology analyses showed that pathways related to ER function and protein misfolding were significantly enriched in patients with FSGS, consistent with activation of the UPR (Navarro-Betancourt et al., 2020).

Levels of BiP were monitored by immunohistochemistry in a cohort of patients with primary membranous nephropathy. Compared to healthy controls, patients with refractory membranous nephropathy showed significantly elevated glomerular expression of BiP, consistent with activation of the UPR (Tao et al., 2016). Expression of CHOP was higher in glomeruli of patients with primary FSGS than in control (Bek et al., 2006). Compared to control biopsies, immunofluorescence staining of CHOP and ATF6 was increased in glomeruli of patients with diabetic nephropathy (Madhusudhan et al., 2015). Finally, glomerular ultrastructure in kidney biopsies of patients with membranous nephropathy revealed a dilated ER in podocytes, an ultrastructural hallmark of ER stress (Cybulsky, 2017). Together, these various observations provide credible evidence for the activation of the UPR in human glomerular diseases, although at this time, the functional importance of the UPR can only be inferred from experimental studies (described below).

## Autophagy in human glomerular diseases

Mutations in core autophagy genes have been rarely implicated in human diseases (Yang and Klionsky, 2020), and any potential repercussions on glomerular function have been eclipsed by profound deterioration of the central nervous system (Zatyka et al., 2020; Collier et al., 2021). Components of the autophagy cascade are transcriptionally upregulated in glomeruli of patients with membranous nephropathy. Compared to microdissected control glomeruli of pretransplant kidney biopsies, glomeruli of patients with membranous nephropathy showed significant upregulation of Atg3 mRNA (Hartleben et al., 2010). The presence of autophagic vesicles in podocytes of patients with minimal change disease was documented by electron microscopy; interestingly, the number of autophagic vesicles in podocytes correlated with albuminuria and extent of foot process effacement (Ogawa-Akiyama et al., 2020). In a different cohort of patients with minimal change disease, the number of autophagosomes (quantified by electron microscopy) correlated positively with the glomerular filtration rate (da Silva et al., 2020).

It has been suggested that primary FSGS may represent an advanced stage of minimal change disease (Maas et al., 2016), and insufficient autophagy may contribute to the progression of glomerular disease. By electron microscopy, podocytes of patients with minimal change disease had significantly more autophagosomes than podocytes of patients with FSGS (Zeng et al., 2014). Interestingly, in repeat biopsies of patients with minimal change disease, podocytes of patients who progressed to idiopathic FSGS had fewer autophagosomes (quantified by electron microscopy) than patients who maintained the minimal change disease status (Zeng et al., 2014). In a different study, podocytes in patients with FSGS also had significantly fewer autophagosomes than podocytes of patients with minimal change disease (da Silva et al., 2020).

Markers of autophagy are evident in diabetic nephropathy. By immunofluorescence microscopy, the presence of LC3 puncta was higher in glomeruli of patients with diabetic nephropathy, compared to control individuals, consistent with increased autophagosome biogenesis (Liu W. J. et al., 2019). Compared to glomeruli of diabetic patients with minimal proteinuria, glomerular deposits of the autophagy substrate p62 were significantly more abundant in patients with diabetic nephropathy and nephrotic range proteinuria; this result implies impaired autophagic flux (Tagawa et al., 2016). Together, these studies support the view that autophagy is activated in human glomerular diseases, but like in the case of the UPR, demonstration of functional importance of autophagy relies on experimental studies.

## UPR in experimental models of glomerular disease

While there is evidence to support activation of the UPR and autophagy in human glomerulopathies, the functional and mechanistic roles of these pathways have been characterized mainly in experimental models. As noted above, podocytes are synthetically active cells; consequently, ER proteostasis can be challenged by secretory overload or disease states that compromise protein folding (Cybulsky, 2017). The role of ER stress and the UPR in animal models of acquired and genetic human glomerular diseases (Table 1) has been reviewed previously (Cybulsky, 2013, 2017; Ricciardi and Gnudi, 2020; Chen et al., 2021; Gomez-Sierra et al., 2021; Li and Chen, 2021). These podocytopathy models have included include membranous nephropathy (passive Heymann nephritis), anti-GBM nephritis and FSGS (adriamycin nephrosis or puromycin aminonucleoside nephrosis). Animals with disease exhibit increases in various ER stress parameters in glomeruli, ultrastructural changes, including dilated ER in podocytes, and disrupted ER function in podocytes (e.g., as reflected by impaired glycosylation and expression of nephrin) (Cybulsky, 2013, 2017). Here we present some more recent experimental studies. The role of the IRE1α UPR pathway is discussed below.

Chemical chaperones (i.e., small molecules that facilitate protein folding in the ER) have been recognized to effectively reduce ER stress and glomerular injury in various experimental models of glomerular disease, including FSGS (Yee et al., 2018), membranous nephropathy (Tousson-Abouelazm et al., 2020), and diabetic nephropathy (Qi et al., 2011; Cao et al., 2016; Wang et al., 2017). Earlier results have been confirmed and extended in more recent studies, and together they support a pathogenic role of ER stress in these glomerulopathies.

Inhibition of pro-apoptotic UPR signals may be a potential mechanistic intervention in certain glomerular diseases associated with podocyte ER stress. The pro-apoptotic transcription factor CHOP is involved in apoptosis in response to persistent ER stress (Iurlaro and Munoz-Pinedo, 2016). Experimental diabetic nephropathy in mice (streptozotocin-induced hyperglycemia) is associated with albuminuria, glomerular mesangial expansion, and increased levels of CHOP (and BiP) in kidney cortex. Global deletion of CHOP attenuated glomerular injury and albuminuria in streptozotocin-induced hyperglycemia (Wu et al., 2010). Recently, inhibition of CHOP translation with intraperitoneal injections of anti-sense oligonucleotides for 8 weeks attenuated glomerular injury and albuminuria in diabetic db/db mice (Shahzad et al., 2021). Treatment was associated with a reduction in apoptotic cells (assessed by TUNEL staining) in renal cortex. However, since human podocytes do not appear to undergo apoptosis in glomerulopathies (Kriz et al., 2013), it remains to be determined if CHOP inhibition would be a useful approach in human disease.

BiP is an ER chaperone, but under conditions of ER stress, BiP can also be expressed at the cell surface. In a study of diabetic nephropathy, treatment of cultured mesangial cells with high glucose induced cell surface BiP expression (Van Krieken et al., 2019). Cell surface BiP interacted with integrin β1 and activated focal adhesion kinase and downstream phosphatidylinositol 3-kinase (PI3K)/AKT signaling, as well as extracellular matrix protein synthesis. Cell surface BiP was upregulated in glomeruli of diabetic mice, suggesting this pathway may mediate a profibrotic response in diabetes.

## Autophagy in experimental models of glomerular disease

In mice, conditional deletion of autophagy mediators disrupts podocyte homeostasis and accelerates glomerular injury (Hartleben et al., 2014; Cybulsky, 2017). Thus, autophagy most likely plays a protective role in the preservation of podocyte proteostasis and glomerular function in normal aging and disease. Furthermore, the UPR and autophagy are complementary proteostasis resources (Rashid et al., 2015), and some studies have shown simultaneous activation of the UPR and autophagy in podocytes. Indeed, the UPR and autophagy may be modulated in parallel by the same UPR transducer. For example, during ER stress in cultured GECs, chemical inhibition or genetic deletion of IRE1α impaired both ER chaperone production and autophagosome biogenesis (Navarro-Betancourt et al., 2020).

Compared to other cell types, it has been suggested that podocytes have high levels of basal autophagy *in vivo*. This conclusion is in part based on studies in transgenic mice expressing green fluorescent protein (GFP)-tagged LC3. These mice show a large number of fluorescent puncta (i.e., autophagosomes) in podocytes (Hartleben et al., 2010); however, it is unclear whether these puncta indicate robust autophagosome biogenesis or a slow autophagic flux (Klionsky et al., 2021). Furthermore, the promoter driving the GFP-LC3 transgene is very active in podocytes compared to other kidney cells, which may contribute to an apparently high number of autophagosomes (Klionsky et al., 2021).

Deletion of Atg5 in podocytes did not affect glomerular development, but aged Atg5 KO mice (8 months) developed albuminuria and upregulation of ER stress markers (Hartleben et al., 2010), providing evidence for the cooperation of the UPR and autophagy in the proteostasis network. Importantly, compared to treated control, non-proteinuric podocyte-specific Atg5 KO mice developed significantly more albuminuria and glomerular injury in experimental FSGS (Hartleben et al., 2010). Thus, the importance of autophagy was revealed via aging or disease. A different approach to delete Atg5 in podocytes and tubular cells produced a more severe phenotype. At 8 weeks, mutant mice had defective glomerular autophagosome biogenesis and developed progressive albuminuria that led to kidney failure by 6 months, with histological changes resembling FSGS (Kawakami et al., 2015).

In models of high fat diet-induced diabetes, albuminuria and glomerular injury were significantly aggravated in mice with podocyte-specific deletion of Atg5, compared to treated control (Tagawa et al., 2016; Yoshibayashi et al., 2020). Proteomic analysis of autophagy-deficient cultured GECs exposed to albumin overload revealed significant upregulation of UPR components (Yoshibayashi et al., 2020), suggesting complementarity of the UPR and autophagy. In rats with experimental membranous nephropathy (passive Heymann nephritis), albuminuria and podocyte loss were associated with glomerular upregulation of autophagy and UPR chaperones (monitored by immunoblotting) (Feng et al., 2014). Pharmacological inhibition of autophagy aggravated albuminuria and foot process effacement in rats with experimental FSGS (puromycin aminonucleoside nephrosis); conversely, stimulation of autophagy with rapamycin attenuated glomerular injury in this model (Zeng et al., 2014).

Studies in cultured cells support the view that autophagy is instrumental for cell survival during ER stress. In cultured GECs treated with tunicamycin or thapsigargin, the addition of a chemical inhibitor of autophagy increased ER stress-induced apoptosis monitored with propidium iodide flow cytometry (Cheng et al., 2015). Likewise, knockdown of Atg7 enhanced apoptosis in GECs exposed to tunicamycin (Feng et al., 2014). Autophagy may also be necessary for basal ER proteostasis, since pharmacologic inhibition of autophagy induced ER stress, as evidenced by upregulation of the UPR transcription factor CHOP (Fang et al., 2014). In tunicamycin-treated GECs, chemical inhibition or genetic deletion of IRE1α impaired the UPR and autophagy, and increased apoptosis (Navarro-Betancourt et al., 2020).

It should be noted that while conditional KO of autophagy mediators in podocytes have demonstrated an important cytoprotective role of autophagy as mice age, the phenotypes of these mice have tended to be mild. IRE1α seems to be dispensable for podocyte function early in life (Navarro-Betancourt et al., 2020; Yoshida et al., 2021). In contrast, a recent study has shown that mice with podocyte-specific deletion of Sel1L (a component of the ERAD pathway) develop severe congenital nephrotic syndrome shortly after weaning and die prematurely (Yoshida et al., 2021). This disruption of ERAD prevented maturation of nephrin, leading to its retention in the ER. ERAD is a quality control mechanism responsible for retrotranslocation of misfolded ER proteins for proteasomal degradation. Although discussion of ERAD is beyond the scope of this review, further studies will be required to define the importance of ERAD in podocyte proteostasis.

## The IRE1α pathway in experimental models of glomerular disease

Global genetic KO of IRE1α (*ERN1* gene) in mice is embryonic lethal (day 12.5 of gestation) due to defects in the placenta (Iwawaki et al., 2009). This severe phenotype may explain why, to our knowledge, mutations in IRE1α have not been demonstrated as causes of human diseases, although certain cancer cell lines contain IRE1α mutations (Greenman et al., 2007; Xue et al., 2011). Accordingly, conditional KO of IRE1α in mice is required to ascertain its function(s) in various organs. Podocyte-specific IRE1α KO mice have no functional or phenotypic abnormalities at a young age; however, aged male mice (i.e., older than 20 weeks) develop progressive albuminuria, loss of podocytes, glomerular collagen deposition, podocyte foot process effacement and ectopic formation of occludens junctions, and impaired glomerular autophagy, as shown by a decreased number of autophagosomes and a reduced glomerular level of LC3-II (Kaufman et al., 2017). This result indicates that the IRE1α axis preserves glomerular function in the context of physiological aging. In contrast, podocyte-specific XBP1 KO mice develop normally for up to a year (Hassan et al., 2016).

**FIGURE 3:**
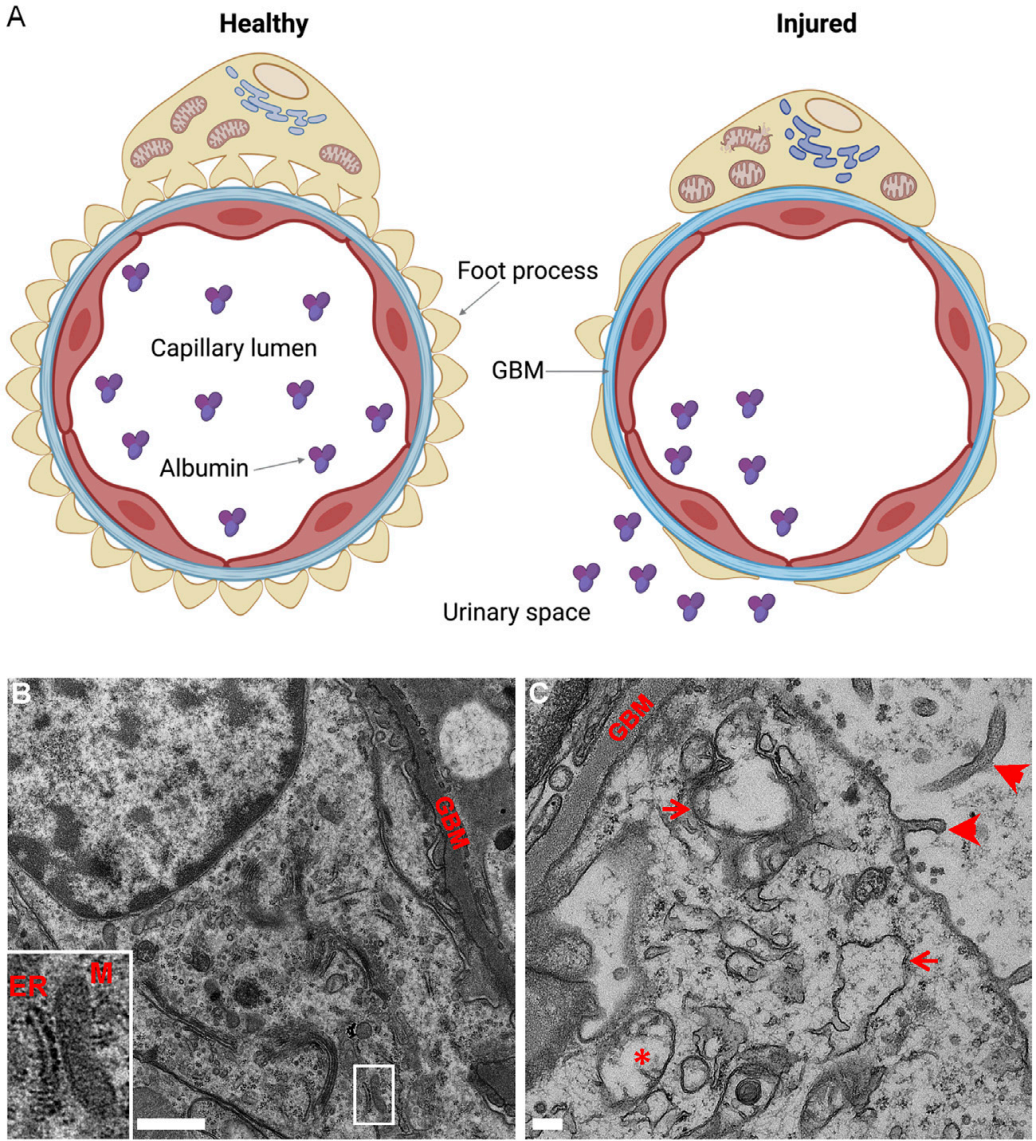
Podocyte-specific deletion of IRE1α exacerbates podocyte injury in adriamycin (ADR) nephrosis. **(A)** Glomerular capillary loop. The glomerular capillary wall consists of podocytes (yellow), GBM (blue) and endothelial cells (red). Healthy podocytes show intact foot processes and organelles, including ER and mitochondria. The glomerular capillary wall is impermeable to albumin. Injured podocytes (e.g., in ADR nephrosis) show effacement of foot processes and albumin passes through the glomerular capillary wall into the urine. Deletion of IRE1α in podocytes exaggerates albuminuria and foot process effacement, and leads to thickening of the GBM, as well as swelling of the ER and mitochondrial damage. **(B,C)** Representative electron micrographs of ADR-treated control and podocyte-specific IRE1α KO mice. **(B)** There is some foot process widening in ADR-treated control mice, although podocyte organelles appear normal. A normal mitochondria-associated membrane is shown in B (inset; M, mitochondrion). **(C)** ADR-treated KO mice show focal foot process effacement, microvillous transformation of podocyte plasma membranes (arrowheads), widening of the GBM, as well as swelling of the ER (arrows) and damage to mitochondria (*) in podocytes. Graphics created with BioRender software; panels B and C are adapted from (Navarro-Betancourt et al., 2020).

The role of IRE1α in the maintenance of proteostasis becomes more evident when cells or organisms are challenged by induction of disease or by an additional stressor. In mice, podocyte-specific deletion of XBP1 together with the Sec63 translocon caused foot process effacement, glomerulosclerosis, and progressive albuminuria starting at 10 weeks (Hassan et al., 2016). The XBP1-Sec63 double deletion appears to have secondarily hyperactivated IRE1α and induced a cytotoxic UPR. In contrast, deletion of IRE1α in podocytes aggravated glomerular injury, podocyte loss, and albuminuria in mouse anti-GBM nephritis, (Kaufman et al., 2017), indicating that IRE1α is necessary for attenuation of acute injury. Furthermore, in experimental FSGS (murine adriamycin nephrosis), podocyte-specific deletion of IRE1α markedly exacerbated albuminuria, foot process effacement, and GBM expansion (Figure 3) (Navarro-Betancourt et al., 2020). Compared to adriamycin-treated control mice, glomeruli of IRE1α KO mice exhibited impaired chaperone production and autophagosome biogenesis. Mechanistically, IRE1α stimulated transcription of Atg5, Atg7, and MAP1LC3B (LC3) genes in response to ER stress (Navarro-Betancourt et al., 2020). Interestingly, after adriamycin treatment, podocytes of IRE1α KO mice showed striking mitochondrial injury, which was absent in podocytes of treated control mice (Figure 3). This intriguing finding points to a connection of the IRE1α pathway with maintenance of mitochondrial integrity (also see below). Indeed, ER-mitochondrial crosstalk has been recently receiving considerable attention (Hasegawa and Inagi, 2020).

IRE1α was reported to be protective in the context of chronic diabetic glomerular disease (Xie et al., 2021). IRE1α signaling was upregulated in glomeruli of db/db mice, and podocyte-specific deletion of IRE1α aggravated albuminuria and morphological glomerular injury in mice with streptozotocin-induced diabetes. Deletion of IRE1α was associated with a decrease in alcohol dehydrogenase-1 expression, although the mechanism of glomerular injury requires further study. Finally, long-term ischemia-reperfusion injury in mice activated the STAT3 pathway and mesangial cells proliferation in glomeruli, caused secretion of glomerular matrix proteins, and promoted glomerular sclerosis. These changes were reduced in mice pretreated with an IRE1α RNase inhibitor, as well as a JNK inhibitor (Liang et al., 2022). The authors suggested that the IRE1α/JNK pathway may be targeted to attenuate transition of acute kidney injury to CKD. Indeed, there is emerging evidence that the IRE1α-XBP1 axis can modulate renal inflammation and fibrosis (Chen et al., 2021).

## Non-canonical functions of IRE1α

Emerging evidence indicates that IRE1α signal transduction through transcriptional and post-translational mechanisms can extend beyond protein folding and degradation, i.e., the classical UPR and autophagy (Hetz et al., 2020). Moreover, IRE1α can physically interact with multiple proteins during the UPR, creating a signaling scaffold that determines the outcome of the UPR (Urra et al., 2020). These so-called non-canonical functions of IRE1α are described below.

### Mitochondrial bioenergetics

Mitochondria are rod-shaped organelles bound by a double membrane; the inner mitochondrial membrane is folded into numerous cristae and is the site where the majority of cellular ATP is produced through oxidative phosphorylation (Herst et al., 2017). The mitochondrial network is dynamic and receives input from multiple organelles and signal transduction pathways (De Stefani et al., 2016; Ploumi et al., 2017). The ER physically interacts with mitochondria through its mitochondria-associated membranes (MAMs) (Figure 3B). These ER domains serve as signaling hubs that coordinate mitochondrial metabolism, structure, and motility (van Vliet et al., 2014). Indeed, mitochondrial function and ER-mitochondria communication are essential for proteostasis (Peric et al., 2016; Hasegawa and Inagi, 2020). Protein folding is an energy demanding process because ATP hydrolysis is necessary for chaperone recycling, so it is advantageous for mitochondrial ATP production to be coupled with the load of misfolded proteins in the ER lumen (Saibil, 2013). Remarkably, IRE1α is enriched at the MAMs and participates in mitochondrial physiology (Mori et al., 2013). Knockdown of IRE1α induced mitochondrial fission (Son et al., 2014); moreover, deletion of IRE1α decreased mitochondrial maximal oxygen consumption and colocalization of the ER with mitochondria (Carreras-Sureda et al., 2019).

The importance of mitochondrial oxidative phosphorylation for podocyte homeostasis is controversial (Gujarati et al., 2020). Resting podocytes have been reported to depend on anaerobic glycolysis for energy generation (Brinkkoetter et al., 2019); on the other hand, mutations in mitochondrial proteins lead to mitochondrial dysfunction, podocyte injury and FSGS, supporting a role for mitochondrial function in maintaining podocyte health (Doleris et al., 2000; Hotta et al., 2001; Antonenkov et al., 2015). Moreover, mitochondrial function is essential for attenuating podocyte injury in the context of glomerular disease (Casalena et al., 2014; Elimam et al., 2016; Elimam et al., 2019). As noted above, in experimental FSGS, IRE1α KO podocytes showed significant mitochondrial injury, compared to IRE1α-replete control. By electron microscopy, podocyte-specific IRE1α KO mice revealed ultrastructural damage, characterized by mitochondrial edema and loss of inner cristae (Navarro-Betancourt et al., 2020) (Figure 3C). Furthermore, in cultured GECs, deletion of IRE1α reduced ATP-linked respiration and the maximal oxygen consumption rate under basal conditions (Navarro-Betancourt et al., 2020). Thus, IRE1α signaling is required to maintain mitochondrial structure or function in healthy and diseased podocytes.

### Metabolism

The class I PI3K pathway integrates signals from multiple receptor tyrosine kinases (Fruman et al., 2017). Upon stimulation by insulin, the insulin receptor activates PI3K at the plasma membrane. PI3K is a heterodimer of a regulatory (p85) and a catalytic (p110) subunit, that when activated, converts phosphatidylinositol 4,5-biphosphate to phosphatidylinositol 3,4,5-triphosphate. This recruits the protein kinase B family, initiating a signaling cascade that promotes glucose utilization and cell growth (Schultze et al., 2012). Following stimulation by insulin, p85 monomers interact with XBP1s to favor its nuclear translocation, and nuclear XBP1s prepares ER proteostasis resources for the challenges of cell growth and nutrient storage (Park et al., 2010; Winnay et al., 2010; Madhusudhan et al., 2015). Interestingly, defects in the nuclear translocation of XBP1s in podocytes are evident in human and murine diabetic nephropathy, and the impaired p85-XBP1s interaction is associated with a maladaptive UPR (Madhusudhan et al., 2015).

The IRE1α pathway may also modulate insulin signaling via post-translational mechanisms. Pharmacological ER stress induced the phosphorylation of insulin receptor substrate-1 at serine 307 by the IRE1α-JNK axis, inhibiting insulin signaling and possibly reducing post-translational modification of clients for the ER (Aguirre et al., 2002; Ozcan et al., 2004). The relevance of this phenomenon in podocytes has not been investigated.

### ER-Golgi trafficking, vesicular transport and reticulophagy

**FIGURE 4:**
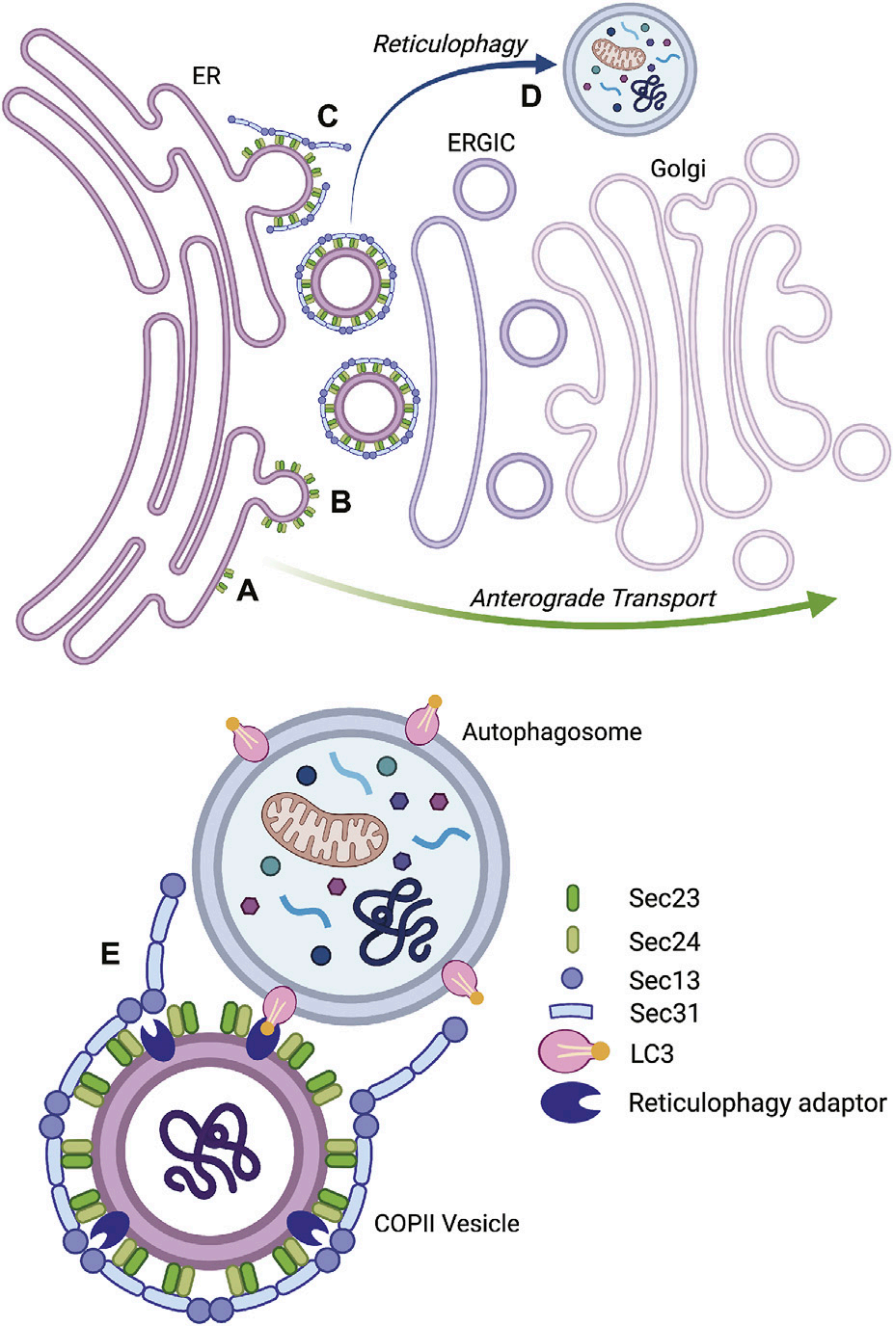
Vesicular transport by the coat protein complex II (COPII) system and reticulophagy. Proteins in the secretory pathway exit the endoplasmic reticulum (ER) in COPII vesicles (left), transit through the ER-Golgi intermediate compartment (ERGIC), and reach the Golgi apparatus (right), where they acquire additional post-translational modifications and are sorted to their final destination. The inner layer of the COPII coat is formed by Sec23-Sec24 heterodimers that assemble at the ER membrane **(A)** and bend it to promote the budding of vesicles **(B)**. The rigid outer cage of COPII vesicles is composed of Sec13-Sec31 heterotetramers **(C)**. During ER stress, some COPII vesicles may be delivered to autophagosomes to undergo reticulophagy **(D)**. This process is assisted by reticulophagy adaptors, such as RTN3L, that recognize LC3 in autophagosome membranes **(E)**. The IRE1α pathway promotes reticulophagy in podocytes. Type IV collagen is produced in the ER and is exported by the COPII system; however in response to ER stress, IRE1α stimulates the expression of Sec23B and RTN3L. Sec23B- and RTN3L-positive ER fragments carry misfolded collagen IV to autophagosomes **(E)**. Reticulophagy relieves the burden of misfolded proteins in the ER and provides cellular membranes for autophagosome elongation. Graphics created with BioRender software.

Activation of the IRE1α-XBP1s axis is a hallmark of secretory cells (Hess et al., 2011; Pramanik et al., 2018; Tsuchiya et al., 2018; Hetz et al., 2020), and recent research has explored the effects of IRE1α in ER protein export. Proteins in the secretory pathway exit the ER in vesicles, transit through the ER-Golgi intermediate compartment (ERGIC), and reach the Golgi apparatus, where they acquire additional post-translational modifications, undergo glycan maturation, and are exported to their final location in cellular membranes or the extracellular space (Huang and Wang, 2017; Makhoul et al., 2019) (Figure 4). More than an intermediate sorting station, the ERGIC is a dynamic cluster of tubulovesicular membranes that participate in autophagy (Ge et al., 2013), protein quality control (Zuber et al., 2001; Hermosilla et al., 2004), and innate immunity (Dobbs et al., 2015).

The earliest step in anterograde transport is mediated by the coat protein complex II (COPII) system, which bends the ER membrane to generate vesicles carrying ER contents (Lord et al., 2013; Peotter et al., 2019). The inner layer of the COPII coatomer is formed by Sec23 and Sec24 heterodimers, while the outer cage is composed of Sec13 and Sec31 heterotetramers (McCaughey and Stephens, 2018) (Figure 4). Indeed, IRE1α-XBP1s transcriptionally upregulates components of COPII vesicles (Shoulders et al., 2013; Taguchi et al., 2015; Bujisic et al., 2017; Liu L. et al., 2019; Navarro-Betancourt et al., 2022), and promotes the synthesis of ER-derived vesicles (Taguchi et al., 2015; Liu L. et al., 2019). Podocytes produce multiple integral and extracellular proteins that transit from the ER to the Golgi apparatus through the COPII system, and then via the secretory pathway. These include nephrin and podocin in the slit diaphragm (Saleem et al., 2002; Roselli et al., 2004), podocalyxin in the apical membrane (Schnabel et al., 1989), and collagen IV in the GBM (Abrahamson et al., 2009). More details on the regulation of ER export and COPII traffic may be found elsewhere (McCaughey and Stephens, 2018; Saraste and Marie, 2018; Peotter et al., 2019).

Interestingly, during stress, ER-derived vesicles from the COPII system may be redirected to autophagy (Li et al., 2020). In fact, nascent phagophores contact ER exit sites (Biazik et al., 2015), and COPII vesicles provide membranes for autophagosomal growth (Shima et al., 2019). Furthermore, deletion of core COPII components impaired autophagy in yeast (Ishihara et al., 2001). In mammalian cells, phosphorylation of the COPII protein Sec23B by Unc-51 like autophagy activating kinase 1 (ULK-1) rerouted COPII vesicles toward autophagosomes, where ER membrane and contents underwent degradation (Jeong et al., 2018). Considering that Sec23B is an IRE1α-dependent protein (Shoulders et al., 2013; Liu L. et al., 2019; Navarro-Betancourt et al., 2022), and that IRE1α is a bona fide regulator of autophagy (Rashid et al., 2015), IRE1α could therefore be involved in autophagy through the export of COPII vesicles. Colocalization of autophagosomes with protein complexes near the ER and Golgi apparatus was documented in podocytes (Narita et al., 2011).

A mechanistic link between ER stress, IRE1α, COPII traffic, and autophagy has indeed been characterized recently. In GECs subjected to ER stress, expression of Sec23B and the reticulophagy adaptor protein reticulon-3-long (RTN3L) was enhanced in an IRE1α-dependent manner (Navarro-Betancourt et al., 2022). ER stress enhanced autophagy via IRE1α, and knockdown of Sec23B reduced autophagosome formation. In addition, ER stress stimulated colocalization of autophagosomes (LC3) with Sec23B and RTN3L in an IRE1α-dependent manner. The functional relevance of this pathway was demonstrated by the observation that during ER stress, GBM α5 collagen IV colocalized with RTN3L and autophagosomes; moreover, degradation of RTN3L and collagen IV increased, and the turnover was blocked by deletion of IRE1α. In experimental FSGS, expression of Sec23B, RTN3L, and LC3-II increased in glomeruli of control mice, but not in podocyte-specific IRE1α KO mice (Navarro-Betancourt et al., 2022). Therefore, during ER stress, IRE1α can redirect a subset of Sec23B-positive vesicles to deliver RTN3L-coated ER fragments to autophagosomes and misfolded collagen IV may be an IRE1α-dependent reticulophagy substrate (Figure 4). Reticulophagy is a novel outcome of the IRE1α pathway in podocytes.

### RIDD in glomerular diseases

Dysregulation of microRNAs is a novel research area in glomerular diseases (Nalewajska et al., 2019). The IRE1α ribonuclease may facilitate autophagy through degradation of an autophagy inhibitor. In response to ER stress, IRE1α can degrade multiple miRNAs involved in autophagy inhibition (Upton et al., 2012), including miR-17 (Duan et al., 2015; Guo et al., 2016), miR-34a (Liu et al., 2014; Liu et al., 2017; Pang et al., 2017), miR-96 (Gan et al., 2017; Shi et al., 2017; Yu et al., 2018), and miR-125b (Cao et al., 2018; Ren et al., 2018; Xiao et al., 2018). Alternatively, the miRNAs cleaved by IRE1α may participate in adaptive processes beyond canonical proteostasis resources. In hepatocytes, IRE1α degrades miRNAs from the miR-200 and miR-34 families to maintain the expression of the ligand-activated transcription factor peroxisome proliferator-activated receptor α (PPARα) and the NAD-dependent deacetylase Sirt1 (Wang et al., 2018). These key regulators of metabolism also have nephroprotective effects (Wakino et al., 2015; Ma et al., 2020). For example, global deletion of PPARα exacerbated glomerular injury and albuminuria in adriamycin nephrosis; conversely, treatment with the PPARα agonist fenofibrate attenuated proteinuria and podocyte foot process effacement in control mice injected with adriamycin (Zhou et al., 2011a). In transgenic OVE26 mice with early onset type one diabetes, podocyte-specific inducible overexpression of sirtuin-1 or treatment with a sirtuin-1 agonist attenuated albuminuria, glomerular oxidative stress, and podocyte foot process effacement (Hong et al., 2018).

### Other roles of IRE1α

Podocyte function relies heavily on a specialized cytoskeleton, and cell division requires a major rearrangement of the cytoskeleton. When podocytes bypass cell cycle checkpoints and attempt cell division, they are unable to complete cytokinesis and detach from the GBM (i.e., mitotic catastrophe) (Lasagni et al., 2013). Indeed, binucleated podocytes with micronucleoli were evident in kidney biopsy electron micrographs from patients with FSGS and collapsing FSGS (Mulay et al., 2013). In response to ER stress, IRE1α may stimulate proliferation of cancer cells through transcriptional upregulation of cyclin A1 by means of XBP1s (Thorpe and Schwarze, 2010). The role of IRE1α in the postmitotic regulation of cell cycle has not been explored.

Upregulation of protein kinase Cα (PKCα) is associated with endocytosis of nephrin and disruption of slit diaphragms in the context of hyperglycemia (Quack et al., 2011). IRE1α may form a complex with PKCα and filamin-A, which leads to phosphorylation of filamin-A by PKCα and stimulation of cell migration. Importantly, dimerization of IRE1α was essential for activation of PKCα, but the kinase or ribonuclease activities were not necessary, and the process occurred in the absence of ER stress (Urra et al., 2018). This observation is consistent with a model where IRE1α molecules cluster as dynamic scaffolds that regulate protein-protein interactions (Urra et al., 2020). Whether IRE1α can participate in remodeling of the slit diaphragm requires investigation.

## Development of UPR and autophagy biomarkers and potential for drug targeting

Given that the UPR has a mechanistic role in podocyte diseases and activation of the UPR is evident before a marked decline in glomerular function, modulation of the UPR could potentially be used to attenuate progression of disease (Cybulsky, 2017; Chen et al., 2021; Gomez-Sierra et al., 2021; Li and Chen, 2021). However, establishment of successful therapies in glomerular disease will depend on selection of appropriate patients, in this case, patients where the UPR is activated. Monitoring components of the UPR in kidney biopsies may be useful for determining if the UPR is activated, although it may be advantageous to monitor the UPR in urine, since such an approach would be non-invasive and would allow for serial monitoring. ER chaperones contain, or may lack, a C-terminal ER retention motif (KDEL). Interestingly, certain ER chaperones that lack KDEL are secreted from podocytes extracellularly and are detectable in urine (Kim et al., 2016; Tousson-Abouelazm et al., 2020; Li and Chen, 2021). The use of urinary ER chaperones as biomarkers has been assessed in experimental models of glomerular injury (Li and Chen, 2021). Rats with podocyte diseases (experimental membranous nephropathy and FSGS) showed increases in various glomerular ER chaperones and increased urinary non-KDEL chaperones, including ERdj3 (DNAJB11) and mesencephalic astrocyte-derived neurotrophic factor (MANF), coinciding with proteinuria (Tousson-Abouelazm et al., 2020). Rats treated with a chemical chaperone to decrease ER protein misfolding showed reduced proteinuria and urinary ER chaperones, compared with vehicle-treated controls. Thus, improved ER protein folding and reduced ER stress can be monitored with urinary ER chaperones. The study showed only a weak correlation between urinary ER chaperone levels and the amount of proteinuria in glomerulopathies, and these chaperones were not secondary to proteinuria-induced tubular ER stress (Tousson-Abouelazm et al., 2020). In another study, mice expressing mutant laminin-β2 in podocytes (nephrosis analogous to Pierson syndrome) excreted MANF into urine, which was detectable before the onset of albuminuria and histologic glomerular injury (Kim et al., 2016; Li and Chen, 2021).

The UPR chaperone ERp57 that contains KDEL was reported to be secreted by podocytes during ER stress and to promote the deposition of extracellular matrix. Secretion was believed to be due to an interaction of ERp57 with extracellular matrix protein(s). ERp57 was absent in the urine of healthy control volunteers and patients with acute kidney injury, but detectable in patients with diabetic nephropathy and microalbuminuria (Dihazi et al., 2013). These results suggest that ERp57 may be a biomarker for progressive fibrotic glomerular diseases.

In regard to potential biomarkers of autophagy, serum levels of beclin-1 were lower in patients with diabetic nephropathy than in control subjects. Interestingly, in the diabetic group, serum beclin-1 showed an inverse correlation with the degree of albuminuria (Naguib and Rashed, 2018), consistent with the notion of autophagy dysfunction in late-stage diabetic nephropathy (Tagawa et al., 2016). The results of these studies are promising, but further research into ER stress biomarkers will be required to establish their clinical utility in identifying ER stress/UPR activation in the glomerulus.

Various drugs have been shown to modulate ER stress pathways in experimental models. These have been reviewed previously (Cybulsky, 2017; Gomez-Sierra et al., 2021; Li and Chen, 2021). As stated above, chemical chaperones can improve ER protein folding or correct misfolding. Other drugs can modulate the activities of UPR transducers or their substrates, increase ER chaperoning capacity, or increase degradation of misfolded proteins. More recently, ER calcium stabilizers (Park et al., 2019) and CHOP anti-sense approaches (discussed above) (Shahzad et al., 2021) were shown to reduce renal injury. Correction of protein misfolding ameliorates cystic fibrosis in humans (Wang and Kaufman, 2016). However, insufficient mechanistic insight into glomerular ER stress has so far limited use of similar approaches in glomerulopathies. With better understanding and monitoring, drugs that target ER stress could enhance therapeutic opportunities in glomerular diseases.

Considering that the outcomes of the IRE1α pathway in podocytes appear to be cytoprotective, and that multiple adaptive effects are mediated transcriptionally, selective activation of the ribonuclease domain of IRE1α could potentially promote proteostasis and reduce injury in glomerular disease (Cybulsky, 2017). For example, some IRE1α kinase inhibitors also block ribonuclease activity (Ghosh et al., 2014), while other kinase inhibitors paradoxically stimulate the ribonuclease (e.g. APY29) (Han et al., 2009). Other drugs directed at IRE1α have been shown to stimulate the IRE1α ribonuclease independently of the kinase (e.g. IXA4, IXA6 and G-9807) (Ferri et al., 2020; Grandjean et al., 2020; Siwecka et al., 2021). Importantly, IXA4 was shown to reduce cytotoxicity by improving folding of destabilized variants of amyloid precursor protein in cultured cells (Grandjean et al., 2020), and IXA4 activated protective IRE1α/XBP1s signaling in the liver and pancreas *in vivo*, mitigating obesity-driven metabolic dysfunction (Madhavan et al., 2022). Thus, stimulation of the IRE1α ribonuclease is an interesting therapeutic approach; however, one must be cautious, since an excessively potent drug effect could potentially hyperactivate IRE1α and result in cytotoxicity (Upton et al., 2012; Iurlaro and Munoz-Pinedo, 2016; Siwecka et al., 2021).

## Summary and conclusions

Podocytes are synthetically-active terminally-differentiated cells. Podocytes produce complex proteins that are critical to the maintenance of glomerular permselectivity; to sustain this role, they require robust ER protein quality control. The ER function of podocytes is perturbed in the course of different glomerular diseases, and podocytes may activate the UPR and autophagy to relieve the consequences of protein misfolding in the ER. Disruption of proteostasis, activation of the UPR, and upregulation of autophagy are evident in human podocytes and in experimental models of glomerular disease. The IRE1α pathway is a major signaling node of ER proteostasis and regulates adaptive resources that facilitate protein folding and degradation. More recent studies point to additional non-canonical functions of IRE1α. Deletion of IRE1α in podocytes disrupts podocyte function in health and disease; however, the mechanisms of IRE1α signaling in podocyte proteostasis and glomerular disease require further characterization. Understanding the molecular basis of glomerular disease and the podocyte proteostasis network may allow the development of mechanistic interventions that preserve podocyte health and glomerular permselectivity.

## Future perspectives

We have highlighted a number of breakthroughs in our understanding of IRE1α signaling and function, but several aspects of IRE1α and XBP1 require further study. The proteostatic role of IRE1α in the glomerulus under resting conditions was more evident in male mice, perhaps due to sex-specific differences in basal intraglomerular hemodynamic pressure or other factors (Kaufman et al., 2017). In future studies, such sex-specific differences will require additional consideration. Signals from IRE1α can lead to both adaptive or cytotoxic outcomes (Chen and Brandizzi, 2013). While the effect of IRE1α on cell fate depends on the kind of injury, duration of the stimulus, and cell type (Karagoz et al., 2019), the regulation of adaptive versus cytotoxic outcomes needs to be further elucidated. Indeed, the precise mechanism of IRE1α kinase and RNase activation remains uncertain. Furthermore, XBP1s can heterodimerize with other transcription factors (Kishino et al., 2017; Vidal et al., 2021), bind to cytoplasmic proteins (Madhusudhan et al., 2015) and undergo post-translational modifications (Lee et al., 2011; Jiao et al., 2017). How these interactions affect downstream responses requires clarification.

Regulation of mitochondrial oxidative phosphorylation is a novel non-canonical function of IRE1α. The contribution of mitochondrial function to podocyte health is controversial and has received considerable interest in recent years (Gujarati et al., 2020). The IRE1α pathway may potentially be involved in ER-mitochondrial interactions and maintenance of mitochondrial energy production in podocytes through the regulation of mitochondrial calcium influx (De Stefani et al., 2016; Carreras-Sureda et al., 2019). It will be crucial to corroborate the functional relevance of podocyte mitochondria to glomerular health and the mechanistic role of IRE1α in the regulation of mitochondrial function in podocytes.

Collagen IV is a complex molecule synthesized in podocytes, and is a critical component of the GBM (Abrahamson et al., 2009). During ER stress, collagen IV misfolding is likely to be enhanced. There is emerging evidence that misfolded collagen IV may be processed via reticulophagy (Navarro-Betancourt et al., 2022). Given the importance of maintaining GBM structure and permselectivity in health and disease, the intracellular turnover and degradation of GBM collagen requires further characterization.

Transcriptomic analyses have provided evidence for ER stress and activation of the UPR in human glomerular disease (Navarro-Betancourt et al., 2020). Collections of kidney tissues from patients with these diseases are increasing, and it may be useful to analyze newer datasets to corroborate findings based on earlier collections (Saez-Rodriguez et al., 2019). Modulation of the UPR could potentially be used as therapy to attenuate the progression of glomerular disease in humans (Cybulsky, 2013, 2017; Gomez-Sierra et al., 2021; Li and Chen, 2021). Given the protective role of IRE1α in experimental glomerulopathies, such as FSGS, the use of selective activators of the IRE1α RNase may be a promising approach (Grandjean et al., 2020; Siwecka et al., 2021). As the next step, these drugs will need to be evaluated in experimental models of glomerular disease. The establishment of successful therapies for glomerular diseases targeting IRE1α or the UPR will depend on the ability to select patients where glomerular pathology is associated with ER stress. Non-invasive biomarker approaches, including the monitoring of UPR components in the urine have shown promise in identifying glomerular ER stress in preclinical studies (Dihazi et al., 2013; Kim et al., 2016; Tousson-Abouelazm et al., 2020; Li and Chen, 2021). The steadily enlarging collections of biological samples from human patients provide opportunities to extend the evaluation of such biomarkers to human subjects.
